# HEROHE Challenge: Predicting HER2 Status in Breast Cancer from Hematoxylin–Eosin Whole-Slide Imaging

**DOI:** 10.3390/jimaging8080213

**Published:** 2022-07-31

**Authors:** Eduardo Conde-Sousa, João Vale, Ming Feng, Kele Xu, Yin Wang, Vincenzo Della Mea, David La Barbera, Ehsan Montahaei, Mahdieh Baghshah, Andreas Turzynski, Jacob Gildenblat, Eldad Klaiman, Yiyu Hong, Guilherme Aresta, Teresa Araújo, Paulo Aguiar, Catarina Eloy, Antonio Polónia

**Affiliations:** 1 I3S—Instituto de Investigação e Inovação em Saúde, Universidade Do Porto, 4200-135 Porto, Portugal; econdesousa@gmail.com (E.C.-S.); jvale@ipatimup.pt (J.V.); pauloaguiar@ineb.up.pt (P.A.); celoy@ipatimup.pt (C.E.); 2 INEB—Instituto de Engenharia Biomédica, Universidade Do Porto, 4200-135 Porto, Portugal; 3 Department of Pathology, Ipatimup Diagnostics, Institute of Molecular Pathology and Immunology, University of Porto, 4200-135 Porto, Portugal; 4 College of Electronic and Information Engineering, Tongji University, Shanghai 201804, China; 1810865@tongji.edu.cn (M.F.); yinw@tongji.edu.cn (Y.W.); 5 School of Computer, National University of Defense Technology, Changsha 410073, China; kelele.xu@gmail.com; 6 Department of Mathematics, Computer Science and Physics, University of Udine, 33100 Udine, Italy; vincenzo.dellamea@uniud.it (V.D.M.); labarbera.david@spes.uniud.it (D.L.B.); 7 Computer Engineering Department, Sharif University of Technology, Tehran 1458889694, Iran; ehsan.montahaei@gmail.com (E.M.); soleymani@sharif.edu (M.B.); 8 Private Group Practice for Pathology, 23552 Lübeck, Germany; turzynski@debitel.net; 9 DeePathology, Hatidhar 5, Raanana 4365104, Israel; jacob@deepathology.ai; 10 Roche Diagnostics GmbH, Nonnenwald 2, 82377 Penzberg, Germany; eldad.klaiman@roche.com; 11 Department of R&D Center, Arontier Co., Ltd., Seoul 06735, Korea; yyhong@arontier.co; 12 INESC TEC—Institute for Systems and Computer Engineering, Technology and Science, 4200-465 Porto, Portugal; guilherme.aresta@gmail.com (G.A.); teresa.safinisterraaraujo@meduniwien.ac.at (T.A.); 13 FEUP—Faculty of Engineering, University of Porto, 4200-465 Porto, Portugal; 14 FMUP—Faculty of Medicine, University of Porto, 4200-319 Porto, Portugal

**Keywords:** breast cancer, HER2, deep learning, computational pathology

## Abstract

Breast cancer is the most common malignancy in women worldwide, and is responsible for more than half a million deaths each year. The appropriate therapy depends on the evaluation of the expression of various biomarkers, such as the human epidermal growth factor receptor 2 (HER2) transmembrane protein, through specialized techniques, such as immunohistochemistry or in situ hybridization. In this work, we present the HER2 on hematoxylin and eosin (HEROHE) challenge, a parallel event of the 16th European Congress on Digital Pathology, which aimed to predict the HER2 status in breast cancer based only on hematoxylin–eosin-stained tissue samples, thus avoiding specialized techniques. The challenge consisted of a large, annotated, whole-slide images dataset (509), specifically collected for the challenge. Models for predicting HER2 status were presented by 21 teams worldwide. The best-performing models are presented by detailing the network architectures and key parameters. Methods are compared and approaches, core methodologies, and software choices contrasted. Different evaluation metrics are discussed, as well as the performance of the presented models for each of these metrics. Potential differences in ranking that would result from different choices of evaluation metrics highlight the need for careful consideration at the time of their selection, as the results show that some metrics may misrepresent the true potential of a model to solve the problem for which it was developed. The HEROHE dataset remains publicly available to promote advances in the field of computational pathology.

## 1. Introduction

### 1.1. Breast Cancer Diagnosis

**Figure 1:**
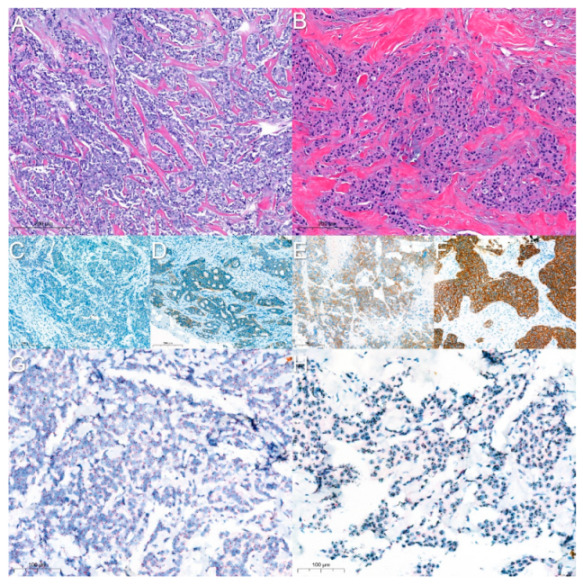
(**A**) HER2-negative BC (HE); (**B**) HER2-positive BC (HE); (**C**–**F**) HER2 IHC (score of 0, 1+, 2+, and 3+, respectively); (**G**,**H**) bright-field ISH assay (HER2-negative and HER2-positive, respectively).

Breast cancer (BC) is the most common cancer worldwide, with more than two million new cases and more than half a million deaths every year, representing roughly 25% of all cancer cases in women [1]. BC detection usually starts with self-checkups via palpation or regular screenings through imaging techniques (ultrasound and/or mammography). When an abnormality is detected, a breast biopsy can be performed, consisting of the sampling of breast tissue through a needle, which is processed and stained with hematoxylin and eosin (HE) to allow visual observation of the tissue under an optical microscope by a medical expert (i.e., a pathologist) (Figure 1A,B) [2].

The microscopic evaluation of BC allows the determination of histological type according to the WHO classification, which, in about 75% of the cases, is invasive carcinoma, not otherwise specified (NOS) [2]. The remaining 25% of the cases are represented by more than 15 special subtypes of BC, some of which are associated with a favorable prognosis, and others with an unfavorable prognosis [3].

All invasive BCs are graded according to histological criteria based on the semi-quantitative evaluation of three morphological features: the amount of glandular differentiation, the degree of nuclear atypia, and the mitotic rate. Each morphological feature is assessed independently with a scoring system of 1 to 3, and the scores are combined to achieve a final histological grade [4]. Several studies have shown that histological grade is an independent prognostic factor in BC, along with lymph node status and tumor size [5].

Finally, current guidelines recommend routine evaluation of ER (estrogen receptor), PgR (progesterone receptor), and HER2 (human epidermal growth factor receptor 2) status in all patients with invasive BC, recurrences, and metastases [6,7]. The evaluation of these biomarkers provides useful predictive information regarding response to targeted therapy.

### 1.2. HER2 Assessment

HER2 is a transmembrane protein receptor with tyrosine kinase activity, being amplified and/or overexpressed in approximately 15% of BC cases [7]. These BC cases are classified as HER2-positive, being associated with aggressive clinical behavior, but also with better responses to HER2-targeted therapies. Several clinical trials have shown an association between these therapies, and a significant improvement in disease-free survival and overall survival for patients with HER2 positivity [8,9,10], thus making the correct identification of this BC subtype of paramount importance.

Usually, HER2 evaluation begins with the analysis of protein expression using specific antibodies that recognize the protein by immunohistochemistry (IHC). In this test, the following results can be achieved: negative (score 0 or 1+), equivocal (score 2+), positive (score 3+), and indeterminate (Figure 1C–F). Equivocal and indeterminate samples require a reflex test, consisting of the evaluation of HER2 amplification with either fluorescence or bright-field in situ hybridization (ISH) assays (Figure 1G,H) [7]. IHC is easier to perform than ISH; however, the latter test is more robust, but also more expensive [11], and can ultimately classify BC samples as HER2-positive and HER2-negative, providing the basis for the application of HER2-targeted therapy.

Typical of most ancillary tests in pathology laboratories, both IHC and ISH tests are sensitive to pre-analytical conditions, such as ischemic time, type of fixative, and duration of fixation [7]. The above-mentioned conditions can compromise the results of the tests, being responsible for the presence of false-negative and false-positive results, which can constitute a major impact on the effectiveness of the implemented treatment.

### 1.3. Digital Pathology

The approval of digital pathology (DP) systems by the US Food and Drug Administration (FDA) has accelerated the implementation of DP in many pathology departments across the globe [12]. There are several advantages described in the literature for using whole-slide images (WSI) instead of glass slides. These include instant sharing of slides for educational purposes or internal/external consultation of challenging cases, as well as for the practice of telepathology [13]. Nevertheless, the main advantage of WSI is the potential for the application of image analysis tools for in silico evaluation that could go beyond traditional quantification analysis, such as IHC analysis, and achieve qualitative analysis to create computer-aided diagnostic (CAD) tools.

### 1.4. Computer-Aided Diagnosis

CAD systems comprise image analysis and machine learning methodologies developed to assist physicians during diagnosis. Their use can not only speed up the diagnostic process, but also increase the accuracy of diagnosis [14,15].

The rise in accessible computing power and large dataset sizes available has allowed neural networks (NNs) to be used in image analysis. NN are networks of transfer functions resembling networks of biologic neurons, hence their name. During the training process, the input weights and internal parameters of each transfer function are adjusted independently to minimize the difference between the output label and the correct label (also known as ground truth). Convolutional neural networks (CNNs) have a specific configuration for identifying and extracting features in images through alternating various convolutional and pooling layers before sending the information (feature map) into a NN. The analysis of the different pathways inside a NN after the training process to understand which features generate a given output is extremely difficult, especially in the case of NNs with multiple deep layers (deep NNs), resulting in these models being known as “black boxes”. The representation of the feature map (saliency map) of CNNs can be overlaid on the original image to highlight the areas of the image the NN is using for the classification, being less opaque than other deep learning methods [16]. NNs can learn practical features directly from the training images by optimizing the classification loss function, opposed to the hand-crafted feature extraction methods. As such, the construction of NNs requires less field knowledge to apply to a given classification system. Despite this, it has been shown that these deep learning methods can reach a greater performance in image classification, including medical images [17,18,19,20,21].

In [22,23], methods for automatic nuclei segmentation and feature extraction were developed, allowing the application of different classifiers to differentiate between benign and malign BC. The more complex three-class problem, discriminating between normal tissue, in situ carcinoma, and invasive carcinoma, was addressed in [24]. In [25], the four-class problem (classifying breast tissue as normal tissue, benign, in situ carcinoma, and invasive carcinoma) was tackled by manually extracting features, while in [17], a deep learning approach was taken. 

The methods described rely on imaging data to classify tissue into two, three, or four classes, but none address the subsequent steps to assess HER2 status in invasive BC. This problem was previously addressed in [26], where the authors described a model for the density counting of fluorescence ISH amplification signals for HER2 status assessment. The model nevertheless requires in situ hybridization to be performed. In [27,28], a deep learning approach was developed to automatically segment cancer cells, and to quantify HER2 expression in IHC images. In 2016, the HER2 Scoring Contest [29] was proposed to compare and advance the state-of-the-art artificial intelligence-based methods to automate HER2 scoring in IHC images. The methods were evaluated against a human consensus ground truth. In addition, this paper reports on a “man versus machine” competition, in which the automated methods outperformed expert pathologists.

The prediction of molecular subtypes in BC was attempted using image analysis of HE and deep learning methods [30]. The work consisted in the evaluation of BC histological images from the Carolina Breast Cancer Study in a tissue microarray (TMA) with molecular classification performed using the PAM50 gene signature. The authors were able to correctly classify high-grade tumors and ER status with accuracies above 80%. The molecular classification accuracy was less impressive (77%), and it was not able to classify the usual four subgroups, but only two larger subgroups (basal versus non-basal subtypes). One of the limitations of this study was the use of TMAs, which, in this case, consisted of just one to four tumor tissue cores per patient of 1mm diameter; this may be an insufficient amount of tumor to be analyzed, and may have compromised the extraction of features for subsequent image classification. Nevertheless, the work provided strong proof of principle that molecular classification can be predicted based on the extraction of HE features. 

The prediction of the expression of molecular biomarkers in breast cancer based only on the evaluation of digitized HE-stained specimens was also attempted by Shamai and colleagues [31]. In this work, a deep convolutional neural network (CNN) based on residual network (ResNet [32]) architecture was developed to predict 19 biomarkers, including ER, PgR, and HER2, from tissue morphology. For these three biomarkers, the areas under the receiver operating characteristic curve (AUC) were 0.80, 0.75, and 0.74, respectively. The data originated from a single institution (Vancouver General Hospital), and included only TMA images from 5356 patients, rather than WSI, representing two important limitations.

Naik and colleagues [33] developed a multiple instance deep-learning-based neural network to predict the same molecular biomarkers from HE-stained WSI. The algorithm, based on ResNet50, was trained using a multi-country dataset of 3474 patients (Australian Breast Cancer Tissue Bank and The Cancer Genome Atlas), and achieved AUCs of 0.92, 0.81, and 0.78, for ER, PgR, and HER2, respectively. 

Following the same rationale, Kather and co-workers [34] developed a deep learning model based on ShuffleNet [35] to predict molecular alterations in 14 of the most common solid tumor types, including breast cancer. The system trained on The Cancer Genome Atlas dataset was able to infer, from histology images alone, at least one mutation in all except one tested tumor type. In breast cancer, ER, PgR, and HER2 subtypes could be predicted, with AUCs of 0.82, 0.74, and 0.75, respectively.

More recently, in 2022, Farahmand and colleagues [36] developed a deep-learning-based model for predicting the HER2 status of tumor regions. By training the model on manually annotated tumor regions, the authors were able to achieve an AUC of 0.90 in a cross-validation protocol, although this value dropped to 0.81 in an independent test set.

### 1.5. HER2 on HE (HEROHE) Challenge

Challenges are excellent opportunities to advance towards state-of-the-art technologies in any given field by gathering experts with different backgrounds to solve one scientific question, and thus promoting a proper balance between competition and collaboration. 

The HEROHE Challenge was developed with the aim of predicting the HER2 status in invasive BC samples via the analysis of HE slides, without access to IHC or ISH assays. Image analysis algorithms for HER2 prediction may not only decrease considerable costs for pathology laboratories, but also serve as safety nets for the typical analysis of HER2 by IHC and ISH. The HEROHE Challenge aimed to promote the creation of image analysis algorithms able to, at least, replace a considerable amount of HER2 tests in BC. This would reduce the costs of pathology exams, accelerate HER2 status determination, and/or pinpoint cases that, despite being deemed conclusive by IHC, could benefit from additional testing to reduce the existence of false-negative or -positive results. This tool could also be used to select the sample most likely to be positive in the case of patients with multiple samples, reducing the cost of analyzing all samples.

Although pathologists rely on IHC and/or ISH assays for the evaluation of HER2 in BC, previous literature shows that HER2-positive BC is associated with different morphological features compared to HER2-negative BC. These features consist of poor differentiation (more solid tumors without tubule formation), higher nuclear pleomorphisms (high nuclear grade), and higher level of mitosis, which are all aggregated in the establishment of histological grade [2]. Many other morphological features might exist to differentiate between these two molecular subtypes of BC, some of which can be subtle or difficult to use through the visual evaluation of the pathologist. Nevertheless, this concept establishes the morphological basis for the success of the proposed task. In addition, previous deep learning models have been used to predict IHC images from the HE slides [37,38,39], thus establishing the computational basis for the success of the HEROHE Challenge.

In this work, we outline the organizational steps of the HEROHE Challenge, the first challenge developed to predict HER2 status from HE-stained WSI, and present the methods and results obtained by the participating teams.

## 2. Materials and Methods

### 2.1. HEROHE Challenge Organization

The HEROHE Challenge was organized as a parallel event of the 16th European Congress on Digital Pathology (ECDP2020). Although the ECDP2020 was canceled due to the coronavirus pandemic, the HEROHE Challenge was performed successfully. The HEROHE Challenge website was hosted on the Grand Challenge servers with the domain https://ecdp2020.grand-challenge.org/ (accessed on 23 May 2022). The Grand Challenge is one the largest platforms for medical imaging challenges, with, at the time of writing, more than 40,000 users. Hosting HEROHE at the Grand Challenge website allowed for an easy setup while maximizing the number of researchers reached. The challenge was also advertised through the social media networks and official webpage of the ECDP2020, and monetary prizes were awarded to the three best-performing methods. 

Unlike previous challenges, where IHC images were part of the training and test datasets, the goal here was to predict HER2 status directly from the morphological features present on the HE-stained images. Thus, the training dataset consisted of 359 WSIs of invasive BC tissue samples stained only with HE, and the corresponding image-wise ground truth classification based on IHC and ISH. The cases did not include annotations such as the location of the invasive carcinoma, and no IHC or ISH slides were provided. The ground truth originated from IHC and ISH tests, resulting in a binary classification (negative or positive). Table 1 summarizes the distribution of IHC scores and HER2 status in the training dataset. One case with a score of 1+ exhibited HER2 amplification by ISH, being classified as HER2-positive. There were 358 female cases and one male case, with ages between 24 and 92 years (median of 58 years old). Cases originated from 22 different laboratories, and all ISH tests were performed at Ipatimup Diagnostics (Portuguese national reference center for HER2). Cases with HER2 heterogeneity were not included in the dataset. All cases were classified by two experienced pathologists (CE and AP) according to the latest American Society of Clinical Oncology/College of American Pathologists (ASCO/CAP) guidelines for BC, scanned at Ipatimup Diagnostics with a 3D Histech Pannoramic 1000 digital scanner at 20x magnification, and saved in the MIRAX file format.

On 1 October 2019, the HEROHE Challenge website and training dataset were released. The test dataset was released on 6 January 2020. In total, 150 WSIs, correspondent to 150 cases, were acquired following the same conditions of the training dataset, including the proportion of positive and negative cases (test dataset distribution was not previously known by the participants). Table 2 summarizes the distribution of IHC scores and HER2 status in the test dataset. The case without IHC score was HER2-positive by ISH. There were 149 female cases and one male case, with ages between 33 and 93 years (median of 57 years old), from 17 different pathology laboratories. All cases from the test and training datasets originated from different patients to ensure independence between datasets.

We were also able to trace 116 cases, from both the training and the test datasets, that showed positive (score of 3+) or negative (score of 0 or 1+) results by IHC, and the corresponding ISH results were obtained either by an internal or external quality control protocol. In these cases, there was only one false-negative case by IHC (mentioned above), providing a sensitivity of 0.98, a specificity of 1.00, a positive predictive value of 1.00, and a negative predictive value of 0.98 for the IHC analysis.

To participate and be eligible for the Challenge’s prizes, at least one member of each competing team should be registered to ECDP2020 and submit, until 28 January 2020, the methods code, the test dataset prediction (hard and soft predictions), and a short method description. The ECDP2020 registration requirement was later removed from the challenge rules due to the cancellation of the congress.

### 2.2. Evaluation

For the ranking of the proposed methods, the F_1_ score, the harmonic mean between precision and recall, was used:

$$F_{1}=\frac{2}{P^{-1}+R^{-1}}=2\times \frac{P\cdot R}{R+P}$$

where $P=\frac{\mathrm{tp}}{\mathrm{tp}+\mathrm{fp}}$ is the precision, $R=\frac{\mathrm{tp}}{\mathrm{tp}+\mathrm{fn}}$ is the recall, tp (true positives) is the number of positive cases classified as positive, fp (false positives), is the number of negative cases classified as positive, and fn (false negatives), is the number of positive cases classified as negative. In addition to the F_1_ score, other metrics were also assessed, namely the area under the curve (AUC), precision, and recall, although these were not considered for the ranking of each competing team. The receiver operating characteristic (ROC) curve is a graphical plot of the true-positive rate (TPR), also known as recall, against the false-positive rate (FPR) at various threshold values. $\mathrm{FPR}=\frac{\mathrm{fp}}{\mathrm{fp}+\mathrm{tn}}$, where tn (true negatives) is the number of cases classified as negative. Since the ROC curve is a two-dimensional curve, to compare methods, the entire curve should be collapsed into one single real number; the most common method to achieve this is to calculate the AUC [40].

### 2.3. Competing Solutions

A total of 21 teams or individual participants submitted their methods until the challenge deadline. Below, we briefly describe the methods proposed by the six best teams, which includes the teams achieving AUCs higher than 0.8.

#### 2.3.1. Team Macaroon

**Figure 2:**
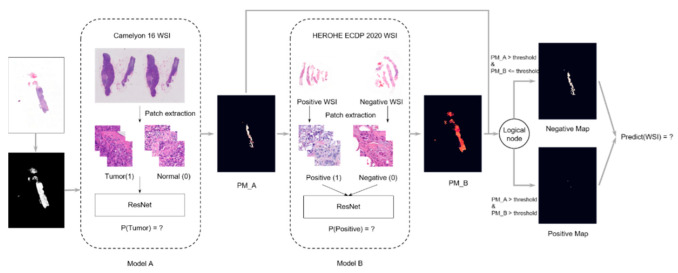
Overall architecture of the model developed by team Macaroon.

The team Macaroon employed a two-stage method to solve the problem (https://github.com/AndrewTal/HEROHE_Macaroon (accessed on 24 July 2022)). In stage A, a ResNet34 model, pre-trained on the CAMELYON16 [19] challenge datasets, was used for training a patch-based (256 by 256 pixels) classification model to differentiate normal tissue patches from tumor patches (see [41] for training details). A probability map, PM_A, was constructed using the results from each WSI. In stage B, each original WSI was down-sampled (ratio 1:2), and a sliding window split it into 256-by-256-pixel patches to be classified by the model from stage A. Potential tumor patches were extracted, and the HER2 status information of the WSI was added to each patch. The resulting dataset was used for training a second ResNet34 model aiming to classify tumor patches as HER2-positive or HER2-negative. Model B was used to generate the final probability map of the WSI (denoted as PM_B). The models were trained using the Adam optimization method with a learning rate initialized as 0.0003, without weight decay. After training, a new WSI is classified as HER2-positive if more than 50% of the tumor patches (those where PM_A > 0.5) are classified as positive by the network B (PM_B > 0.5), and is classified as negative otherwise. The overall architecture of the resulting model is in Figure 2.

#### 2.3.2. Team MITEL

**Figure 3:**
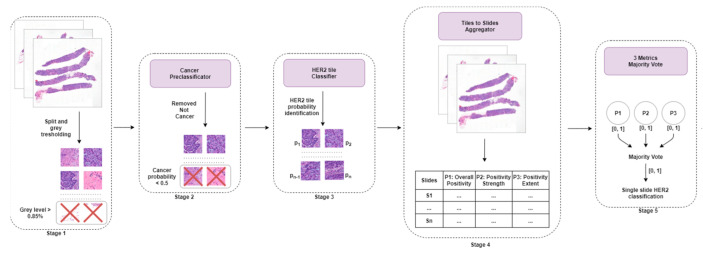
Overall architecture of the model developed by team MITEL.

A five-stage procedure was used by team MITEL (see Figure 3). The full method is described in [42]. A pre-processing step was implemented where each WSI was first down-sampled (ratio = 1:2), and tiles were created by a sliding window of 512 by 512 pixels. Only tiles with an average grey level of <85% were retained. In the second stage, Tumor Detection, tiles were classified as tumor or normal tissue by DenseNet201 [43]. The model used was pre-trained on ImageNet [44], and then fine-tuned on the BACH [18] dataset for the tumor classification task. Tiles classified as normal tissue were discarded, while the others were used in the following stage. In the third stage, HER2 Classification, the remaining tiles were fed into ResNet152 (optimized for precision), and the model was trained to predict the probability of a given tile being from an HER2-positive WSI. In the fourth stage, results from all tiles of any given WSI were aggregated into three WSI-level features:

1. Overall positivity: mean positivity probability for all tiles in a WSI. If above 0.5, the slide is positive for HER2;
2. Strength of positivity: mean positivity probability of positive tiles only. If above 0.66, the slide is positive;
3. Extent of positivity: percentage of positive tiles. If 35% of the tiles for each slide are positive, then the slide is positive.

Finally, in the fifth stage, each WSI was classified via majority voting based on the results of the three conditions. The software and trained models are available online at https://github.com/MITEL-UNIUD/HE2HER2/ (accessed on 20 July 2022).

#### 2.3.3. Team Piaz

**Figure 4:**
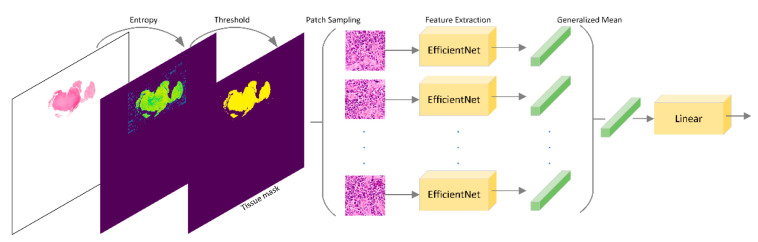
Overall architecture of the model developed by team Piaz.

The team Piaz used a four-stage procedure (https://github.com/IAmS4n/HEROHE (accessed on 23 May 2022)) to classify each WSI in a multi-instance learning fashion (see Figure 4). In stage one, the Shannon entropy of the WSI was computed to identify the most informative regions, and then a threshold, based on the method of minimum value in histogram [45], was found to construct a tissue mask. Next, 256 random patches of 222 by 222 pixels were extracted from the tissue mask’s valid regions at maximum resolution. In the second stage, EfficientNetB0 [46], pre-trained on the BACH challenge [18] dataset, was used and retrained on the HEROHE dataset to extract a 64-dimensional feature array for each patch. The features were extracted using max pooling of the last CNN layer of EfficientNet. In addition, there was a batch norm layer, followed by an absolute operation after each max pooling event. In the third stage, a novel pooling function was developed to aggregate the arrays resulting from each WSI into a single 64-dimensional feature array. A different exponent (denoted as p) of a generalized mean function was used for each feature in a spectrum that varies between p=1, when the generalized mean is equal to the arithmetic mean, and p=16, when the generalized mean will approximate the maximum function. In mathematical terms, let $f_{ij}$ be the jth extracted feature of the ith patch, the jth element of the output vector is ${\left(\frac{1}{n}\overset{n}{\underset{i=1}{\sum }}f_{i,j}{}^{p_{j}}\right)}^{1/p_{j}}$, where $p_{j}=1+15\left(j-1\right)/63$. Finally, in the fourth stage, the WSI classification probability was computed using a linear layer followed by a sigmoid layer on the features resulting from the aggregated 64-dimensional array.

In the test phase, to decrease the patch sampling effect, the result of each WSI was evaluated 64 times, and the final probability is the mean of these values.

#### 2.3.4. Team Dratur

**Figure 5:**
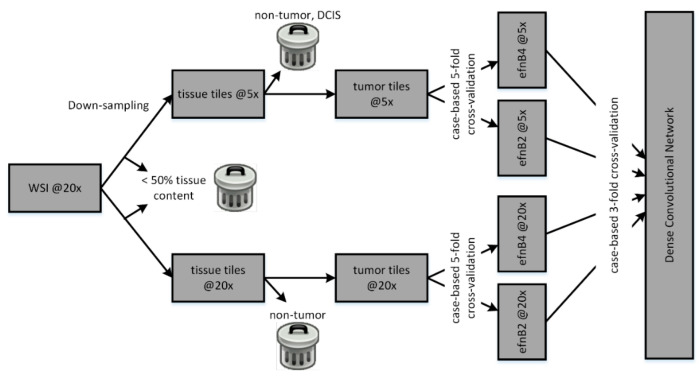
Overall architecture of the model developed by team Dratur.

The team Dratur used a method consisting of two parallel tracks (@20x and @5x tracks) to classify each WSI (see Figure 5) (for details see https://doi.org/10.5281/zenodo.6900746 (accessed on 25 July 2022)). Tumor regions were manually annotated in 3DHistech CaseViewer, and exported to TIFF file format using 3DHistech SlideConverter. After pre-processing for brightness adaption, the TIFF files were sliced with a sliding window procedure, generating 256-by-256-pixel tiles at the original 20× magnification, and 256-by-256-pixel tiles at 5× magnification. Tiles with less than 50% of tissue pixels were discarded. A sample of tissue tiles was manually grouped for vital invasive carcinoma and non-tumor (including ductal carcinoma in situ, DCIS, in the @5× track). Two EfficientNetB4 models were trained to enrich vital invasive tumors in both tracks. Strong and complex data augmentations were applied in the training of all convolutional neural networks, including the modification of hue and saturation, the addition of salt and pepper artifacts, color noise, and block artifacts using the image library (https://github.com/aleju/imgaug (accessed on 23 May 2022)), as well as affine augmentations from the Keras library. HER2-positive and -negative cases were split into five partitions, keeping the class balance in each partition. EfficientNetB4 and B2 models were then trained using a five-fold cross-validation procedure to predict the HER2 status. The resulting soft predictions were fed into a small dense convolutional network (two hidden layers with 32 and 16 nodes, L2 regularization, and drop out) trained with a three-fold split for cross-validation. The models were trained using the Adam optimization method with a fixed learning rate of 0.001. The resulting soft predictions were tested against the training dataset ground truth, and a threshold of 0.47 was defined to generate the hard prediction for each WSI, resulting in the correct classification of 86.39% of the training WSI, compared to a correct classification of 85.27% at a threshold of 0.5.

#### 2.3.5. Team IRISAI

**Figure 6:**
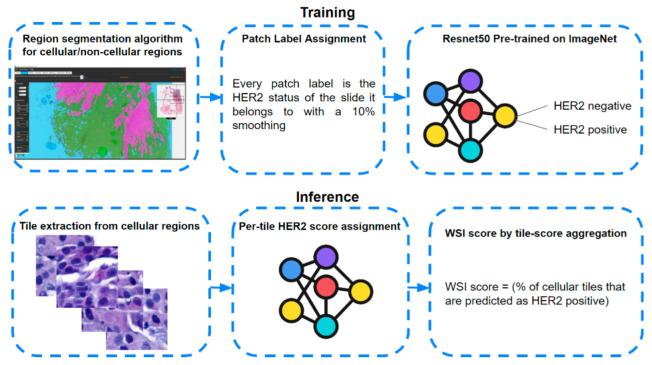
Overall architecture of the model developed by team IRISAI.

The team IRISAI used a two-stage model to solve the problem (see Figure 6). In the first stage, a U-Net [47] model was trained from scratch on slides at 5× magnification, to segment each WSI into “cellular” and “non-cellular” regions. Training images for this task resulted from an interactive set of annotations and corrections performed using DeePathology STUDIO software. In the second stage, 900,000 image patches of size 256 × 256 pixels were extracted from the cellular regions at 20× magnification and used as training dataset. A patch was considered cellular if at least 95% of its pixels were predicted as cellular by the U-Net network of stage one. Patches for the weakly supervised task were labeled by assigning to each one a value according to the corresponding WSI, with a smoothing of 10% to accommodate for patch selection errors. In other words, patches originating from a positive WSI were labeled as 0.9, while those originating from a negative WSI were labeled as 0.1. Standard data augmentation was then applied to the resulting dataset, and a Resnet50 classifier, pre-trained on ImageNet [44], was trained to predict the patch-level labels using an Adam optimizer with default parameters. Finally, the WSI-level HER2 score was computed by splitting it into tiles and measuring the ratio of cellular tiles in that slide that had an output of above 0.5 from the Resnet50 classifier (https://github.com/jacobgil/irisai_herohe (accessed on 22 July 2022)).

#### 2.3.6. Team Arontier_HYY

**Figure 7:**
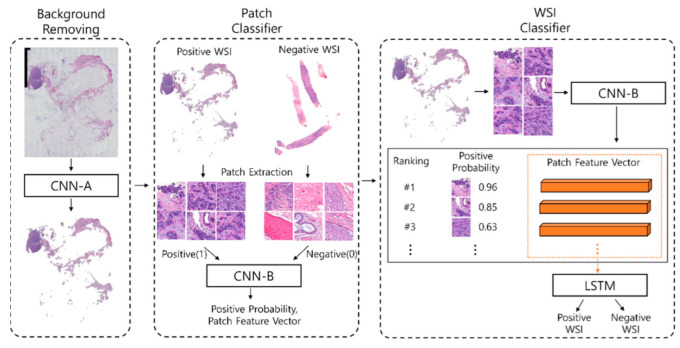
Overall architecture of the model developed by team Arontier_HYY.

The team Arontier_HYY employed a three-stage method to predict HER2 status (see Figure 7). In the first stage, EfficientNetB3 (denoted as CNN-A) was trained from scratch to classify image patches of 1024 by 1024 pixels as “tissue” or “background”. The training dataset for CNN-A consisted of image patches labeled as “background” if they include regions with stains or adipose tissue, as well as if they contain severely blurred regions and patches with less than 25% of tissue (pixel intensities below 240); otherwise, they were classified as “tissue”. After discarding images classified by CNN-A as “background”, in the second stage, a model consisting of two parallel EfficientNets (denoted as CNN-B) was used to extract a feature vector and a score representing the probability of a given tissue patch being from an HER2-positive WSI. In one of the parallel paths, EfficientNetB1 was trained on around 250,000 image patches of 480 by 480 pixels, while in the other, EfficientNetB5 was trained on around 100,000 image patches of size 912 by 912 pixels. Tissue patches were labeled as 0 whenever they originated from an HER2-negative WSI, and as 1 otherwise. Data augmentation was then implemented to generate a more robust model, and the resulting dataset was used to train CNN-B. To minimize class imbalance and inter-WSI variations, within each mini batch, all patch images originated from different WSIs, and 50% were from randomly selected positive WSIs, while the remaining were from randomly selected negative WSIs. In stage three, all feature vectors of any given WSI were sorted in decreasing order by the corresponding score of CNN-B, and fed into a Long-Short Term Memory (LSTM) network with two recurrent layers and one dropout layer, to assess the final WSI prediction.

All patch images were extracted at 20× magnification. All networks were trained around five epochs using the RAdam optimization method with a fixed learning rate of 0.001 and a cross-entropy loss function; batch size for stage two was 64, and for stage three batch size was 1. Five-fold cross-validation was applied, and the final result was an ensemble of the all models that selected based on the highest AUC at the WSI-level for each fold of validation data (https://github.com/arontier/HEROHE_ECDP2020 (accessed on 21 July 2022)).

## 3. Results

The HEROHE Challenge was open for approximately 4 months, between 1 October 2019 and 28 January 2020. During this period, 863 participants registered and had access to the training and test datasets. The labels of the test set were only revealed at the end of the challenge, after the teams submitted their prediction scores and the ranking of the challenge was known. In total, 21 teams or individual participants submitted their results, and F_1_ scores were evaluated to assign the challenge’s final ranking. Table 3 summarizes the evaluation metrics of the teams, including F_1_ score, AUC, precision, and recall.

Two teams developed methods that, by construction, did not quantify the probability of a WSI belonging to one class (i.e., did not infer a soft prediction). The model developed by the team Macaroon classified each WSI after comparing the number of patches classified as tumor by one network and as positive by the second network. Thus, an approximation was needed to compute the AUC. The soft predictions were set to 1 whenever a WSI was classified as positive, and to 0 otherwise. The model developed by team MITEL used a majority voting to assign a class to each WSI. Considering that the proposed method exported three soft predictions, the developers chose to consider, as a representative soft prediction, the prediction resulting from the overall positivity, because it covered all the WSI patches, thus being considered the most exhaustive among the three.

Evidently, the choice of threshold impacts the $F_{1}$ score. The magnitude of this impact was assessed, for each team, by varying a particular threshold from 0 to 1 by steps of 0.01, and, for each threshold value, re-classifying all WSIs in the test dataset and computing the corresponding $F_{1}$ score. Each WSI was classified as positive if the submitted soft prediction was greater than or equal to the threshold, and negative otherwise. The $F_{1}$ score was then evaluated based on the updated classifications. For each team, a theoretical maximum was obtained. Table 4 summarizes the ranking of the teams according to the F_1_ score resulting from the use of the updated threshold. The results reveal that three of the top four teams could have achieved better performances, and the resulting ranking would have changed if different thresholds were chosen. The maximum $F_{1}$ scores for these teams were: (a) Piaz: $F_{1}$ = 0.73 for a threshold of 0.39; (b) MITEL: $F_{1}$ = 0.70 for a threshold of 0.37; and (c) Dratur: $F_{1}$ = 0.69 for a threshold of 0.34.

**Figure 8:**
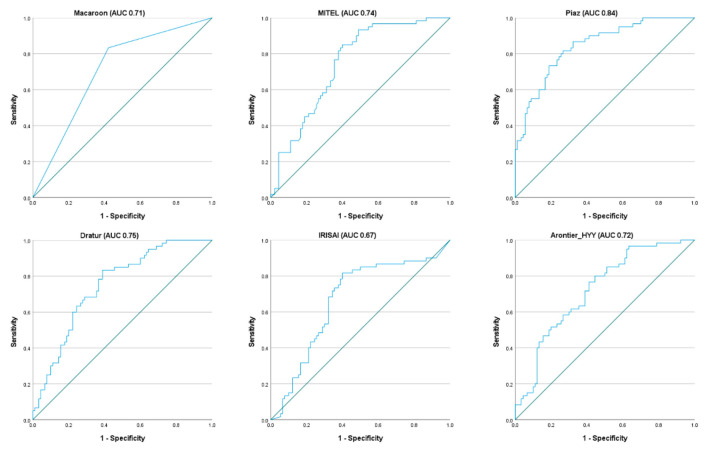
ROC curves for the methods proposed by the six best teams in the test dataset.

**Figure 9:**
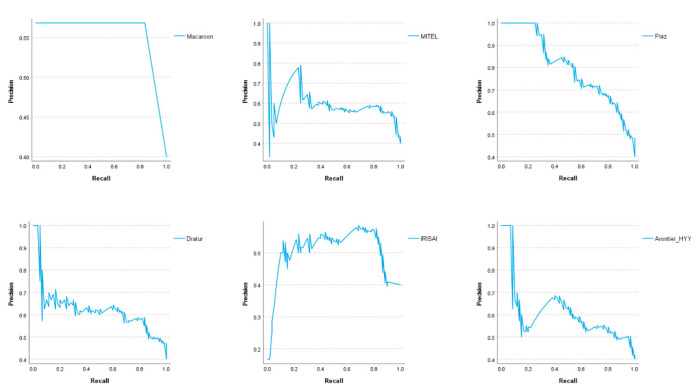
Precision–recall curves for the methods proposed by the six best teams in the test dataset.

Although the $F_{1}$ score was the ranking metric, other metrics were also assessed. The precision and recall were assessed to compute the $F_{1}$ score, while the AUC was measured to allow comparisons to other recently published methods for HER2 prediction [31,33,34,36]. Two teams (MITEL and Dratur, 2nd and 4th place, respectively) achieved AUC results for HER2 prediction similar to those presented in [31,33,34], while the team Piaz (3rd place) achieved the highest AUC of 0.84. Figure 8 and Figure 9 show the ROC analysis with ROC curves and precision–recall curves, respectively, for the methods proposed by the six best teams in the test dataset.

Considering the distribution of cases among the four possible HER2 scores of the IHC test, the precision, recall, AUC, and $F_{1}$ score were also evaluated in the subset of equivocal IHC cases (53 HER2-negative and 32 HER2-positive cases with scores of 2+ in the IHC test; see Table 2). Table 5 summarizes the ranking of the teams according to F_1_ score in the subset of equivocal cases by IHC (score of 2+).

**Figure 10:**
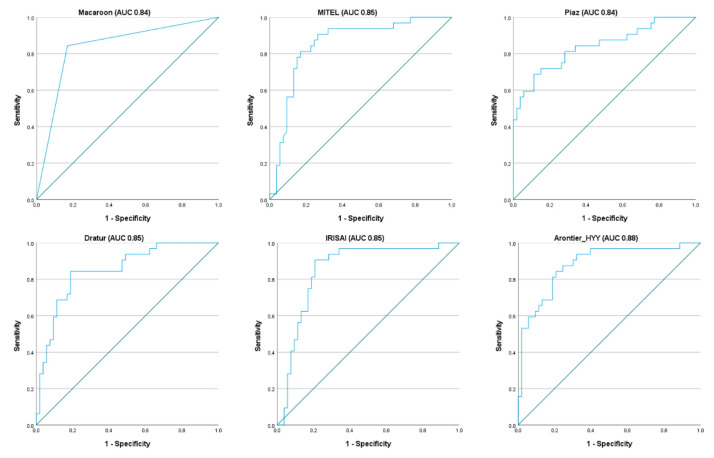
ROC curves for the methods proposed by the six best teams in the subset of equivocal cases by IHC (score of 2+).

**Figure 11:**
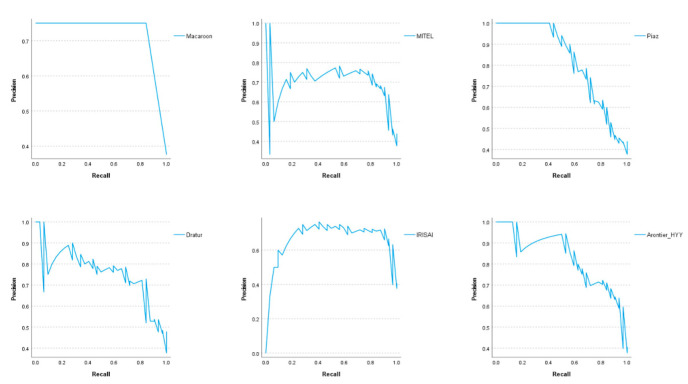
Precision–recall curves for the methods proposed by the six best teams in the subset of equivocal cases by IHC (score of 2+).

Six teams achieved, in this subset, AUCs equal to or greater than 0.84, higher than the AUCs achieved by the models presented in [31,33,34]. Moreover, team Arontier_HYY achieved an AUC of 0.88, similar to the AUC achieved in [36] for cross-validation, and higher than that achieved in an independent test set. Figure 10 and Figure 11 show the results of the ROC analysis, presented as ROC curves and precision–recall curves, respectively, for the methods proposed by the six best teams in the subset of equivocal cases by IHC (score of 2+).

Although the core methodologies differ between the submitted models, some procedures are common among approaches. For example, 20 out of the 21 teams developed methods taking advantage of deep neural networks in one or more steps of their models, and used Python as the main programming language. Reza Mohebbian was the only team that developed a “classical” machine learning model (without deep neural networks), coded on MATLAB. Teams HEROH, HEROHE_Challenge, Institute of Pathology Graz, and katherandco, despite having used Python as the main programing language, also used QuPath [48] in some steps of their methods. Another common step was the split of each WSI into smaller patches. Only three teams used the entire WSI as the input: Reza Mohebbian, who developed a non-deep-learning-based model; Institute of Pathology Graz, who combined a hand-crafted feature extractor developed on QuPath with a custom CNN for classification; and aetherAI, who adapted ResNet50 [32] to produce, as inputs, WSIs resized to a 10,000-by-10,000-pixel canvas. Among, the 20 teams that used deep neural networks in their methods, 12 chose to rely on models pre-trained on other publicly available datasets, ImageNet [44] being the most widespread, although other datasets were also used [18,19,49,50]. Table 6 summarizes the main characteristics of the submitted methods, including the approach method, use of pre-training and external datasets, ensemble size, and size of the images.

## 4. Discussion

As in all challenges, the definition of the metric to access the final ranking is of paramount importance. All metrics that may have been considered to evaluate the methods assess different aspects of the results, thus, they produce different ranks. Since our dataset was imbalanced, metrics such as accuracy (the percentage of total cases correctly classified), which is often used to evaluate classifiers, are not reliable descriptors of the model’s ability to solve the problem at hand. For example, a model that predicts all cases as negative achieves an accuracy equal to the proportion of the negative cases, which, for highly imbalanced datasets, could result in higher classifications. Other metrics are less prone to the class imbalance problem. Among them, precision and recall are good choices; nevertheless, both these metrics fail in some extreme scenarios. The proper balance between these two metrics can be achieved by combining them into a single and more robust metric, the $F_{1}$ score, which only achieves values close to 1 if both the precision and the recall are simultaneously close to 1.

The organizers did not opt for other performance metrics, such as the AUC, to better simulate the clinical practice. Indeed, AUC is a global performance metric that does not necessarily encode the behavior of a system at different regions of the ROC curve. For instance, in clinical practice, it may be of interest to operate on a region of lower or higher FPR, depending on the goal of the screening. However, two models with the same AUC may exhibit different behaviors in these two extreme regions. With this in mind, it was decided to instead ask participants to select a prediction threshold that would maximize the $F_{1}$ score. The main goal was to force the system to form an absolute/objective decision regarding the sample being assessed, serving as a clear second opinion about the patient’s HER2 status, i.e., the participants were asked a priori to select the operation point of their system. 

Previous studies addressing the problem of predicting the expression of molecular biomarkers in breast cancer [31,33,34,36] reported the AUC as a primary performance metric. Consequently, despite having considered the $F_{1}$ score as the ranking metric for the challenge, the AUC was also used here as a metric for performance assessment. The models presented in Section 2.3. Competing Solutions corresponded to those that achieved AUC > 0.8 in Table 3 or Table 5. Although models here presented are compared against other models in the literature [31,33,34,36], it is important to acknowledge that, because the evaluations were performed in different datasets, these comparisons are only indicative, and are not evidence that the models presented here are better or worse than those mentioned above. The comparison between the rankings in Table 3 and Table 5 shows that some methods performed better on some data, and less well on others. For example, team Piaz ranked 3rd when the entire test dataset was evaluated, but its rank dropped to 7th when only the equivocal cases were considered.

An important aspect to be considered by any researcher after training a neural network is to decide on the threshold that will determine the final prediction. If the teams had chosen different thresholds to generate their final predictions, their resulting $F_{1}$ score could have been better. For instance, with this test dataset, the model developed by the team Piaz could have achieved an $F_{1}$ score of 0.73 (instead of the actual 0.64) if the threshold was set to 0.39 (instead of the used 0.5), thus being ranked at the top of the leaderboard. Of course, the value 0.39 was obtained as the best choice for the test dataset, which, by definition, was not available to teams. Nevertheless, the training dataset, or part of it (e.g., validation dataset), should be used to fine-tune the threshold. It is worth noting that, even if the optimum threshold was not the one resulting by the evaluation of the model performance on the training dataset, others close to 0.39 (thresholds between 0.36 and 0.43) would also generate a final $F_{1}$ score greater than 0.70. 

The comparison between the result that team MITEL reached ($F_{1}=0.67$) and the one that they would have had if they used the overall connectivity as the only evaluation feature ($F_{1}=0.70$ for a threshold of 0.37) reveals that, sometimes, a more complex model does not outperform a simpler one. 

The results obtained by the teams in the subset of equivocal cases (Table 5) reveal that most of the top-ranked teams (eight of the top ten, including the top seven teams) achieved better results on this subset than in the whole test dataset (Table 3). For example, team Macaroon achieved an $F_{1}$ score of 0.68 for the whole test dataset, but this rose to 0.79 when considering just the equivocal cases. This difference is due to the increase in precision, which increases from 0.57 to 0.75, while the recall increases from 0.83 to 0.84. Similar changes occurred with other top-ranked teams (e.g., Dratur’s $F_{1}$ score rose from 0.63 to 0.73, Arontier_HYY’s $F_{1}$ score rose from 0.61 to 0.73). Equivalently, the AUC increased in most of the teams. While on the full test dataset only one team achieved an AUC greater than 0.8 (team Piaz with AUC=0.84), on the subset of the equivocal cases, six teams had an AUC above 0.8. The difference between the results achieved for these two groups of cases can be explained by the distribution of cases per HER2 score on both the training and test datasets, with a majority being classified as equivocal on the IHC test (about 60%). This result suggests that HER2-equivocal cases with HER2 amplification are morphologically different from HER2-positive cases by IHC (score of 3+). Indeed, there are studies showing that not only are these cases morphologically different, but the latter group shows a higher proliferative index, higher levels of HER2 amplification, and higher response rates to target therapy [53,54,55,56]. The fact that equivocal cases are the cases sent for reflex ISH analysis, an expensive specialized technique, provides the best performance in these cases of major clinical relevance.

Following a common trend in medical image analysis, most of the teams (20 out of 21) used at least one deep neural network on their models. Given the complexity of the task, many teams split the problem into more than one step, and relied on the combination of more than one deep network to classify each WSI (Table 6). Since the training dataset was comprised of 359 cases, 12 teams chose to use models pre-trained on other datasets. This allowed them to train deeper networks, with up to millions of parameters, potentially improving performance while reducing development and training time. Although most of the models presented here rely on networks pre-trained with other datasets, with the data collected in this challenge, the authors could not find statistical evidence to support that the use of external datasets was a determining factor in the final ranking. As shown in Table 6, the top-ranked teams used models pre-trained with external datasets, but so did some of the lowest-ranked teams (e.g., the first- and last-place teams, Macaroon and UC CSSE, used the CAMELYON dataset as an external source). A study comparing the performance of a larger number of models would be needed to provide a definitive answer to this question. Another common technique was splitting the entire WSI into small tiles (18 out of 21 teams), applying the HER2 classifier to each tile, and later combining the information to get the WSI classification. Most teams (16 out of 21) decided to prune the WSI first by applying a segmentation algorithm that identifies regions of interest (e.g., the two top-ranked teams applied deep learning (DL) algorithms to identify tumor regions while the third-ranked team relied on non-DL algorithms to segment tissue regions) and only then using those regions in the classification step, thus reducing the computational costs, while focusing the WSI classification on targeted regions.

In terms of clinical application, ideally, the next step would be to not only predict the HER2 status in BC samples, but also predict the response of the patients to HER2-targeted therapy. Previous literature shows that morphological clues can be found in the tumor tissue, such as the presence of tumor-infiltrating lymphocytes (TILs), which can be good predictors for HER2-targeted therapy. BC samples with a high number of TILs will more often display complete pathological responses in the surgical specimen after neoadjuvant (before surgery) HER2-targeted therapy and subsequent better disease-free survival [57,58]. Moreover, there might be additional features that could be extracted for predicting the response to HER2-targeted therapy. 

In specific settings of breast cancer, gene expression tests have already been recommended to assess the risk of recurrence and guide oncologists in the difficult decision to use chemotherapy [59,60]. These tests are very expensive and tissue destructive, two major limitations that decrease their use in clinical practice. Furthermore, it has been shown that nuclear morphology features, such as nuclear shape and architecture, can be extracted from HE stained images to predict risk categories using the gene signatures [61]. Combining clinical information with HE stained images of HER2-positive patients has also shown potential for predicting BC recurrence and metastasis [62]. This type of research and prediction can take computational pathology to a level never experienced in medicine.

It would also be clinically relevant to understand why some models perform better than others, and to identify the features that contribute most to predicting HER2 status. Unfortunately, although deep-learning networks perform better than humans in several domains, the complex and opaque black box nature of these networks limits its interpretability [63,64].

In the HEROHE Challenge, it was decided to consider datasets with cases without HER2 heterogeneity since it is the most frequent situation, and to avoid the introduction of unnecessary noise into the training and test datasets. Additionally, heterogeneous cases would require specific tumor annotations. HER2 heterogeneity corresponds to tumors with both HER2-negative and HER2-positive areas, representing a minority of situations (up to 1% of the cases), with patients requiring at least 10% of HER2-positive areas in the BC to be elected for targeted therapy [7]. Although there are morphological features more likely to be associated with HER2-positive BC (as discussed above), making the distinction between HER2-positive and HER2-negative theoretically possible, in cases with HER2 heterogeneity the different areas in the BC appear to be very similar, at least to the pathologist’s assessment [65]. Finally, the proportion of cases in each IHC score was biased towards the equivocal cases (score of 2+). This decision was because these are the cases that require further assessment, namely, by the evaluation of HER2 amplification by ISH, and thus considered by the challenge organizers as the most important cases. Future research to address this problem should consider these aspects.

Roughly, 15% of BC cases are HER2-positive. Nevertheless, the datasets released here failed to follow the real ratio between positive and negative cases. This decision was taken based on the logistics required to release a dataset according to this ratio that still had enough WSI per class. Such a dataset would require more than 3TB of disk space, and would result in a dramatic increase in the communication time required to upload and download all the data. On one hand, such a huge dataset could eventually prevent some teams from participating due to the computational resources that would become necessary. On the other hand, more data may have resulted in better models, although the higher class imbalance could also present different challenges to teams during model development and training.

The rules of the challenge required each team to submit the code of the method, the prediction of the test dataset, and a brief description of the method. In hindsight, the challenge organizers acknowledge that participants should have been asked for more data, namely learning curves, which are now impossible to obtain. Without the learning curves of the individual teams, we cannot know whether a particular model was trained for enough epochs or, on the contrary, whether it was trained for more epochs than it should have been. Problems such as underfitting or overfitting could be identified if the submission of learning curves was mandatory. However, it is important to note that each epoch, considering tiling and data augmentation, consisted of training the model with hundreds of thousands or even millions of images; thus, the smaller number of epochs may not result in a poor model, but instead avoid overfitting. More data would be needed to test this assumption.

## 5. Conclusions

The HEROHE Challenge was developed with the primary goal of promoting the development of computer-aided diagnostic tools to predict the HER2 status in invasive BC samples. Despite the complexity of the proposed task, 21 models were presented, combining different techniques, from standard image analysis to state-of-the-art DL algorithms, and promising results were achieved.

Given the biased distribution of training and test datasets, with most cases classified as equivocal by the IHC test, this work also presented the AUC and $F_{1}$ score of the proposed models in a subset of the test dataset with only equivocal cases (Table 5). Most of the teams performed better in this subsample. Six teams achieved AUCs greater than or equal to 0.84, outperforming the results presented in recently published studies. Team Arontier_HYY achieved the highest AUC (0.88) for this dataset, and the top $F_{1}$ score rose from 0.69 to 0.79 (team Macaroon). This fact suggests that some of the presented models identified features on the HE slides scored of 2+ on the IHC test that can be used to predict HER2 status, something human experts are not able to do. The achieved results are not perfect, and more data may lead to an improvement in the performance of the models, especially in cases scored as 0, 1+, or 3+ by the IHC test that were under-represented in the challenge datasets, and eventually in the equivocal cases as well.

The importance of the metric defined to assess the models’ performance was shown to have a great impact on the final rank. In this work, the importance of a proper selection of the final threshold to separate positive and negative cases was also emphasized by presenting an example revealing that with a better choice of the threshold, the same algorithm would result in a model with a significantly better performance. It was also pointed that the choice of the network, hyperparameters, and all the features of a model has to be carefully evaluated during the development, as well as later, taking into consideration the evaluation of the model on the training and validation datasets. The difference between the result of the team MITEL compared to the result they could have achieved with a simpler model is a detail that is important to keep in mind.

Although the challenge is now closed, the website and datasets will remain public for research purposes, thus further contributing to the development of novel solutions to this field. These solutions may originate from practical clinical problems, such as quality control, assisting in the identification of false-positive or false-negative results, or aiming to increase the understanding of HER2-positive BC, through detailed morphological assessment of the tumor.

## Figures and Tables

**Table 1:** Distribution of IHC scores and HER2 status in the training dataset.

IHC Score	HER2-Negative	HER2-Positive	Total
0	43 (12%)	0 (0%)	43 (12%)
1+	46 (13%)	1 (0%)	47 (13%)
2+	126 (35%)	104 (29%)	230 (64%)
3+	0 (0%)	39 (11%)	39 (11%)
Total	215 (60%)	144 (40%)	359 (100%)

**Table 2:** Distribution of IHC scores and HER2 status in the test dataset.

IHC Score	HER2-Negative	HER2-Positive	Total
0	19 (13%)	0 (0%)	19 (13%)
1+	18 (12%)	0 (0%)	18 (12%)
2+	53 (35%)	32 (21%)	85 (57%)
3+	0 (0%)	27 (18%)	27 (18%)
Not Tested	0 (0%)	1 (1%)	1 (1%)
Total	90 (60%)	60 (40%)	150 (100%)

**Table 3:** Final classification of the HEROHE Challenge according to $F_{1}$ score.

Rank	Team	AUC	Precision	Recall	F_1_ Score
1	Macaroon	0.71	0.57	0.83	0.68
2	MITEL	0.74	0.58	0.78	0.67
3	Piaz	0.84	0.77	0.55	0.64
4	Dratur	0.75	0.57	0.70	0.63
5	IRISAI	0.67	0.58	0.67	0.62
6	Arontier_HYY	0.72	0.52	0.73	0.61
7	KDE	0.62	0.51	0.75	0.61
8	joangibert14	0.66	0.48	0.78	0.60
9	VISILAB	0.63	0.51	0.73	0.60
10	MIRL	0.50	0.40	1.00	0.57
11	aetherAI	0.66	0.49	0.67	0.57
12	NCIC	0.63	0.52	0.62	0.56
13	biocenas	0.57	0.46	0.53	0.50
14	HEROH	0.59	0.46	0.53	0.49
15	Reza Mohebbian	0.61	0.51	0.43	0.47
16	mindmork	0.63	0.53	0.38	0.45
17	Institute of Pathology Graz	0.63	0.50	0.38	0.43
18	katherandco	0.44	0.44	0.40	0.42
19	QUILL	0.63	0.50	0.33	0.40
20	HEROHE_Challenge	0.48	0.37	0.27	0.31
21	UC-CSSE	0.47	0.31	0.27	0.29

**Table 4:** Classification of the HEROHE Challenge when the best possible threshold for the test dataset is used.

Rank	Team	Threshold	F_1_ Score
1	Piaz	0.39	0.73
2	MITEL	0.37	0.7
3	Dratur	0.34	0.69
4	irisai	0.39	0.68
5	Macaroon	0.01	0.68
6	Arontier_HYY	0.17	0.66
7	visilab	0.1	0.65
8	KDE	0.26	0.63
9	katherandco	0.83	0.62
10	QUILL	0.23	0.62
11	aetherAI	0.17	0.6
12	HEROH	0.12	0.6
13	joangibert14	0.5	0.6
14	biocenas	0.23	0.59
15	Institute_of_Pathology_Graz	0.42	0.59
16	mindmork	0.07	0.59
17	NCIC	0.49	0.59
18	Reza_Mohebbian	0.01	0.58
19	uc_csse	0.02	0.58
20	HEROHE_Challenge	0	0.57
21	MIRL	0	0.57

**Table 5:** Classification of the HEROHE Challenge in the subset of equivocal cases by IHC (score of 2+).

Team	AUC	Precision	Recall	F_1_ Score
Macaroon	0.84	0.75	0.84	0.79
Arontier_HYY	0.88	0.67	0.81	0.73
MITEL	0.85	0.74	0.72	0.73
Dratur	0.85	0.71	0.75	0.73
IRISAI	0.85	0.72	0.72	0.72
KDE	0.77	0.67	0.75	0.71
Piaz	0.84	0.79	0.59	0.68
VISILAB	0.77	0.64	0.66	0.65
NCIC	0.70	0.58	0.69	0.63
Biocenas	0.71	0.61	0.63	0.62
AetherAI	0.77	0.53	0.72	0.61
QUILL	0.78	0.79	0.47	0.59
Joangibert14	0.70	0.46	0.72	0.56
MIRL	0.50	0.38	1.00	0.55
Reza Mohebbian	0.64	0.52	0.47	0.49
Institute of Pathology Graz	0.70	0.50	0.47	0.48
HEROH	0.63	0.46	0.50	0.48
Mindmork	0.61	0.43	0.31	0.36
Katherandco	0.32	0.67	0.25	0.36
UC-CSSE	0.61	0.42	0.31	0.36
HEROHE_Challenge	0.50	0.37	0.22	0.27

**Table 6:** Main characteristics of the submitted methods. “Approach” lists the main methods used to classify the WSI; “pre-trained” indicates whether the transfer learning approach was used; “ensemble” indicates whether the method uses one or multiple models, and their number; “external sets” indicates external datasets used in pre-trained models; “input size” indicates the size, in pixels, of the images or tiles required by the model (WSI signifies that the entire WSI was input into the model at the same time).

Rank	Team	Approach	Pre-Trained	Ensemble	External Sets	Input Size
1	Macaroon	ResNet34	yes	2	CAMELYON16	256 × 256
2	MITEL	DenseNet201 + ResNet152	yes	2	ImageNet + BACH	512 × 512
3	Piaz	EfficientNetB0	yes	x	BACH	222 × 222
4	Dratur	EfficientNetB2 + EfficientNetB4 + Custom dense model	yes	5	ImageNet	256 × 256
5	IRISAI	U-Net + ResNet50	no + yes	2	ImageNet	256 × 256
6	Arontier_HYY	EfficientNetB1 + EfficientNetB3 + EfficientNetB5 + LSTM	no	4	x	1024 × 1024 + 480 × 840 + 912 × 912
7	KDE	Custom + InceptionV3	no	3	x	128 × 128
8	joangibert14	ResNet101	yes	x	[49]	224 × 224
9	VISILAB	SE-ResNet50	no	x	x	299 × 299
10	MIRL	DenseNet201	yes	x	ImageNet	9192 × 9192
11	aetherAI	Custom based on ResNet 50 v2	no	x	x	WSI re-scaled to 10,000 × 10,000
12	NCIC	ResNet101 + ResNet50 [51]	yes	2	ImageNet	1024 × 1024
13	biocenas	Custom CNN model	no	3	x	32 × 32
14	HEROH	ResNet18 + ResNet50	yes	2	ImageNet	128 × 128
15	Reza Mohebbian	Custom (non-Deep Learning)	no	x	x	WSI
16	mindmork	Kmeans + U-Net + Xception [52]	no	3	x	256 × 256
17	Institute of Pathology Graz	QuPath for color deconvolution and feature extractor + Custom CNN	no	2	x	WSI
18	katherandco	QuPath for tumor segmentation + ResNet50	no	x	ImageNet	512 × 512
19	QUILL	SuperPixel patch splitting + DenseNet + Mean Shift Clustering	no	2	x	WSI
20	HEROHE_Challenge	Custom CNN + Kmeans + XGBoost	yes	3	CIFAR-10 dataset	200 × 200
21	UC-CSSE	Xception + DenseNet169 + ResNet34 + ResNet101 + random forest + extra trees + gradient boosting	yes	7	CAMELYON16 + Data Science Bowl 2018	299 × 299

## Data Availability

Publicly available datasets were analyzed in this study. This data can be found here: https://ecdp2020.grand-challenge.org (accessed on 23 May 2022).
